# Blood Glucose Control Strategy for Type 2 Diabetes Patients With COVID-19

**DOI:** 10.3389/fcvm.2020.593061

**Published:** 2020-10-28

**Authors:** Hiroyuki Futatsugi, Masato Iwabu, Miki Okada-Iwabu, Koh Okamoto, Yosuke Amano, Yutaka Morizaki, Takashi Kadowaki, Toshimasa Yamauchi

**Affiliations:** ^1^Department of Diabetes and Metabolic Diseases, Graduate School of Medicine, The University of Tokyo, Tokyo, Japan; ^2^Laboratory for Advanced Research on Pathophysiology of Metabolic Diseases, The University of Tokyo, Tokyo, Japan; ^3^Department of Infectious Diseases, The University of Tokyo Hospital, Tokyo, Japan; ^4^Department of Respiratory Medicine, The University of Tokyo Hospital, Tokyo, Japan; ^5^Department of Orthopaedic Surgery, The University of Tokyo Hospital, Tokyo, Japan; ^6^Department of Prevention of Diabetes and Life-Style Related Diseases, The University of Tokyo, Tokyo, Japan; ^7^Toranomon Hospital, Tokyo, Japan

**Keywords:** COVID-19, diabetes mellitus, antidiabetic agents, healthy diet, exercise

## Abstract

Since December 2019, coronavirus disease 2019 (COVID-19) caused by a novel coronavirus has spread all over the world affecting tens of millions of people. Another pandemic affecting the modern world, type 2 diabetes mellitus is among the major risk factors for mortality from COVID-19. Current evidence, while limited, suggests that proper blood glucose control may help prevent exacerbation of COVID-19 even in patients with type 2 diabetes mellitus. Under current circumstances where the magic bullet for the disease remains unavailable, it appears that the role of blood glucose control cannot be stressed too much. In this review the profile of each anti-diabetic agent is discussed in relation to COVID-19.

## Coronavirus Disease 2019 Pandemic

Coronavirus disease 2019 (COVID-19), caused by coronavirus SARS coronavirus 2 (SARS-CoV-2), was originally identified in Wuhan, China in December 2019 (1). Since then, the disease has spread all over the world at a tremendous rate and the number of confirmed cases already exceeded 13 million, killing more than 570 thousand people (2). Though measures are being taken in affected countries, such as lockdown of major cities, to control the pandemic, the numbers are still growing day by day (3, 4).

SARS-CoV-2 belongs to the Betacoronavirus genus as SARS coronavirus (SARS-CoV) and MERS coronavirus (MERS-CoV) do (5). As with SARS-CoV, SARS-CoV-2 spike glycoprotein interacts with and binds to human angiotensin-converting enzyme 2 (ACE2) when the virus enters into target cells (6). While ACE2, a type I transmembrane glycoprotein, serves as a functional receptor for SARS-CoV-2, ACE2 is also shown to play a protective role against acute respiratory distress syndrome (ARDS) and SARS pathogenesis by catalyzing angiotensin I and angiotensin II to angiotensin (1-9) and angiotensin (1-7), respectively (7). However, its overall impact on COVID-19 remains to be further elucidated.

A clue could be found with angiotensin-converting enzyme inhibitors (ACE inhibitors) and angiotensin II receptor blockers (ARBs). Since several data suggested that these agents can increase ACE2 expression through their influence on the level of angiotensin II, there was concern over their potential negative influence on COVID-19 morbidity, severity and mortality rates (8). However, multiple large-scaled studies show that their use affected none of these rates (9–11). Actually, at present, almost all medical associations, including the International Society of Hypertension, American College of Cardiology, American Heart Association and Heart Failure Society of America, have published statements recommending the continued use of ACE inhibitors and ARBs (12, 13).

COVID-19 is thought unlikely to become severe in a majority of cases. In fact, a recent meta-analysis of 47,344 patients with COVID-19 in China shows that the risks of severity and mortality were 18.0 and 3.2%, respectively, among these patients, while these rates increased if patients had comorbidities (14).

## COVID-19 and Diabetes

Diabetes mellitus is another global pandemic affecting 463 million people worldwide (15). In the face of the COVID-19 pandemic, two facts need to be taken to heart. First, nutrition and exercise therapy represent the cornerstone of diabetes management (16). However, the resultant need for home confinement to control the pandemic, as well as for a new lifestyle to reduce the risk of infection, is shown to reduce physical activity and increase sweet food consumption (17, 18), while, now more than ever, the importance of healthy diet and exercise needs to be stressed. Second, which concerns the main theme of this review, diabetes is among the comorbidities associated with increased risk of COVID-19 (19). According to a recent meta-analysis, COVID-19 patients who had diabetes at baseline had increased severity and mortality (HR, 2.11 [CI, 1.40–3.18], 1.69 [CI, 1.22–2.33], respectively), although the prevalence of diabetes in the affected population did not seem to differ from that in the non-affected population in Asia (20). Fortunately, however, it is indicated in a retrospective multicenter study conducted in China that proper blood glucose control may reduce not only the severity of COVID-19 but mortality from the disease in patients with pre-existing diabetes showing improvements in systemic inflammation as measured by serum inflammation markers (21). Actually, in the study, during the 28-day observation period, patients with favorable glycemic control (3.9–10 mmol/L) had a significantly lower mortality rate compared to those with poorly glycemic control (the lowest BG level, >3.9 mmol/L or the highest BG level, >10 mmol/L) (HR 0.14 [CI, 0.03–0.60])(21). In the current situation where COVID-19 and diabetes are so prevalent that they combine to affect a high proportion of patients and where there is no critical medicine or vaccine for COVID-19, the fact that blood glucose control has a role to play in reducing the severity of the disease as well as mortality from the disease, cannot be stressed too much (21).

## Strategy for Blood Glucose Control

Then, how should we achieve blood glucose control? First, we would suggest that PCR-confirmed asymptomatic type 2 diabetic patients with COVID-19 or those with mild self-limiting COVID-19 should continue with their current prescription because, to date, there is no evidence to suggest that certain glucose-lowering agents interact with or worsen the disease in patients with asymptomatic or mild COVID-19 (22). This strategy is supported by the fact that hyperglycemia itself is likely to lead to greater severity of COVID-19 and higher mortality from the disease (23, 24). In patients with moderate to severe symptoms who need hospital admission, however, given the pathophysiological and clinical characteristics of COVID-19, some drugs may not be deemed favorable, due to their side effects that could potentially adversely affect the course of the illness. In this review focused on biguanides, thiazolidinediones, sulfonylureas, dipeptidyl peptidase-4 (DPP4) inhibitors, glucagon-like peptide-1 receptor (GLP-1R) agonists, sodium-glucose cotransporter-2 (SGLT2) inhibitors and insulin, their profiles will be discussed in relation to COVID-19.

## Biguanides

Biguanides, represented by metformin, is one of the most frequently prescribed oral glucose-lowering agents. Metformin mainly functions by activating AMP-activated protein kinase (AMPK) through inhibition of the respiratory chain of mitochondria thereby subsequently reducing gluconeogenesis in the liver (25). In most situations, metformin is a well-tolerated drug with a relatively low rate of adverse effects. However, as it inhibits mitochondrial respiration and increases lactate production, it may induce lactic acidosis in some patients receiving it, with nearly half of all patients developing lactic acidosis dying from it. Of note, while the risk of lactic acidosis is increased in patients with renal or hepatic impairment, dehydration, shock, hypoxic states, sepsis and advanced age (26), these conditions are often found to be present in patients with severe COVID-19 (27). In addition, up to half of all hospitalized COVID-19 patients are shown to suffer deep venous thrombosis (DVT) thus often requiring the use of contrast-enhanced CT for DVT assessment (28). When transient renal impairment occurs following injection of an iodinated contrast agent in patients receiving a biguanide, however, the renal excretion of the drug is decreased, and their lactic acid levels increased, thus placing these patients at risk of lactic acidosis (29). On the contrary, a recent retrospective study performed in China showed that metformin-treated patients hospitalized for COVID-19 had a lower mortality rate compared to non-metformin-treated patients (30, 31). Thus, overall, while diabetic patients with asymptomatic or mild COVID-19 may continue current metformin therapy and further interventional studies should be conducted to prove or disprove this recommendation, it appears that, as a rule, metformin should be withdrawn in hospitalized patients.

## Thiazolidinediones

Thiazolidinediones are shown to achieve their blood glucose-lowering effect by activating peroxisome proliferator-activated receptor γ (PPARγ) thereby increasing insulin sensitivity (32). In addition to their glucose-lowering effects, thiazolidinediones are also shown to exert immunomodulatory effects (33). Given that immune hyperactivity is considered to be involved in the pathophysiology of COVID-19, it appears reasonable to assume that they may have some positive impact on the disease progression (33–36). However, there are also some concerns. First, while its clinical impact is not known, pioglitazone has the potential to enhance ACE2 expression in the liver, adipose tissue and skeletal muscle (37, 38), suggesting that the use of thiazolidinediones may affect not only the prevalence of COVID-19 but the mortality from the disease. Second, thiazolidinediones also act on the collecting tubule to increase water and sodium reabsorption by enhancing the expression of the epithelial Na^+^ channel, thus causing edema and fluid retention (39, 40). This adverse effect may be enhanced in patients with COVID-19, given that the disease is sometimes shown to damage the kidneys and myocardium (41, 42). Therefore, it appears that, for the time being at least, it is advisable to avoid using thiazolidinediones in hospitalized patients.

## Sulfonylureas and Glinides

Sulfonylureas, the oldest oral antidiabetic drugs, are shown to promote insulin release from pancreatic β cells by binding to and closing the ATP-sensitive potassium channel resulting in depolarization of the plasma membrane and increased calcium influx thus leading to insulin exocytosis (43). Again, glinides represent viable options in managing postprandial hyperglycemia due to their rapid and short-lasting insulinotropic effects (44, 45). However, sulfonylureas are known to cause hypoglycemia at a non-negligible rate with the risk shown to increase in acute settings (46), thus making their use inappropriate in patients with severe COVID-19.

## Dipeptidyl Peptidase-4 Inhibitors

DPP4 inhibitors exert their anti-diabetic effects by inhibiting DPP4, which degrades incretin hormones, gastric inhibitory polypeptide (GIP) and glucagon-like peptide-1 (GLP-1), thus elevating blood levels of these hormones. Since these hormones stimulate insulin release glucose-dependently, DPP4 inhibitors lower blood glucose levels with no significant risk of hypoglycemia (43). Although DPP4 is considered to be a functional receptor of MERS-CoV (47), a sibling of SARS-CoV-2, there is no evidence to date to show that it interacts with SARS-CoV-2. Actually, a case control study conducted in Italy showed no association between the exposure to DPP4 inhibitor and the risk of hospitalization due to COVID-19 (48). Of note, DPP4 inhibitors exert anti-inflammatory effects without increasing the risk of infectious disease and thus may prove protective against COVID-19-induced lung injury (49–51). To confirm these hypotheses, a phase 4 clinical trial of linagliptin vs. insulin is currently underway to compare their effectiveness not only in achieving glucose control but in preventing the progression of COVID-19 in type 2 diabetic patients with mild to moderate COVID-19 (52). While, given its glucose-dependent effects and the risk of hypoglycemia thought to be relatively low with this DPP-4 inhibitor, the use of DPP-4 inhibitors as a class may be deemed relatively safe in these patients mild to moderate COVID-19, consideration should be given to switching to insulin in patients with severe COVID-19.

## Glucagon-Like Peptide-1 Receptor Agonists

While the mechanism of action of GLP-1R agonists is not fully understood, it seems likely that it involves cAMP signaling pathways and intracellular glucose metabolism in restoring β-cell glucose sensitivity (53). Due to this mechanism, GLP-1R agonists are assumed to lower blood glucose with relatively low risk of hypoglycemia (54). In addition, they help reduce food intake and body weight and thus may often be beneficial for diabetic patients who tend to be overweight (53–55). Also, their cardio- and renoprotective profile may prove beneficial for patients with COVID-19 (56, 57). In addition, of the GLP-1 agonists, liraglutide has the potential to increase lung ACE2 expression, while the net effect of ACE2 on COVID-19 still remains unknown (58). Nevertheless, GLP-1R agonists may as well be withdrawn from diabetic patients requiring hospitalization with COVID-19, given that their frequent adverse events, gastrointestinal symptoms (e.g., nausea, diarrhea or vomiting) are likely to worsen dehydration and, as a consequence, cause renal failure, which often occur in patients with COVID-19 (27, 53).

## Sodium-Glucose Cotransporter-2 Inhibitors

Unlike other oral glucose-lowering agents described above, this class of drugs exert their effects, independently of insulin, by blocking sodium-glucose cotransporter 2 (SGLT2) in the renal proximal tubules from reabsorbing filtered glucose, i.e., by increasing glucose excretion thereby decreasing levels of blood glucose (59). Moreover, SGLT2 inhibitors are known to have cardio- and renoprotective effects (60–62) and have the potential to improve systemic metabolism, thus possibly preventing respiratory failure and organ dysfunction associated with COVID-19. To test this speculation, a phase 3 international, multicenter, double-blind, randomized, placebo-controlled trial of dapagliflozin is now underway in hospitalized, mild-to-moderate COVID-19 patients with preexisting comorbidities (63). However, an international expert panel warns that increased glucose excretion may also lead to fluid loss, possibly resulting in worsening of dehydration and onset of diabetic ketoacidosis in diabetic patients with COVID-19 (22, 64). Based on these findings, therefore, consideration should be given to discontinuing SGLT2 inhibitors in diabetic patients with COVID-19 at high risk of respiratory failure and thrombosis.

## Insulin Analogs

The only hormone available to lower blood levels of glucose, insulin is released from pancreatic β cells sensitized by glucose influx through glucose transporter type 2 (GLUT2) and stimulates the uptake of carbohydrates, peptides and lipids by other cells. Simultaneously, it inhibits hepatic gluconeogenesis and glycolysis, thus causing a rapid drop of blood glucose (65, 66). In normal settings, the goal of insulin therapy is to reproduce physiologic insulin secretion using long-acting analogs as basal insulin release and rapid-acting analogs as prandial insulin release (67). Unlike other oral antidiabetic agents, insulin has been used in critically ill patients whose prognosis is shown to improve to a greater extent with conventional insulin therapy than with intensive insulin therapy (68, 69). While insulin therapy prior to admission was shown to be associated with higher mortality in patients with COVID-19 (70, 71), there is a possibility that blood glucose control with insulin therapy during hospitalization leads to reductions in the risk of sever disease in these patients (72). Of course, when using insulin, close monitoring of blood glucose levels is essential, because it is sometimes associated with hypoglycemic events thus possibly raising mortality rates in critically ill patients (73, 74). However, given the current circumstances where there is no accumulated evidence to support the use of other agents, insulin appears to be the best choice for diabetic inpatients with COVID-19.

## Ongoing Studies

While the possible harms and benefits of antidiabetic drugs have been summarized in the context of COVID-19, some of these recommendations remain rather hypothetical, because of the paucity of current evidence. Again, several clinical trials are now underway to investigate the actual effect of these drugs in controlling blood glucose diabetic patients with COVID-19, with some of these drugs expected to prevent exacerbation of COVID-19 even in non-diabetic patients. The characteristics of these trials are summarized in Table 1. These include NCT04341935 and NCT04371978, both randomized open label studies in diabetic patients with COVID-19, with the former intended to prove the efficacy of the investigational drugs in controlling blood glucose, and the latter aimed to reveal the efficacy of the study drugs in improving the severity of the disease (52, 75); NCT04365517, also a randomized controlled open label study designed to investigate the potential respiratory role of the DPP4 inhibitor sitagliptin in diabetic patients suffering from pneumonia due to COVID-19 (76). Through these studies, DPP4 inhibitors may be shown to be effective in controlling blood glucose in diabetic patients with COVID-19. Again, a non-randomized matching cohort study, NCT04473274 is intended to investigate the safety of the thiazolidinedione pioglitazone in patients with relative hyperglycemia requiring hospital admission due to COVID-19 (77) and may be able to address the speculative concern posed above. NCT04510194 is a large-scale, randomized, quadruple blinded study evaluating metformin not only for its therapeutic but for its preventive role against COVID-19 (78). Given the large sample size and the reliable study design, as well as the fact that, to date, virtually no drug has been proved to prevent the infection itself, the results of this study are worth paying attention to. Furthermore, another large-scale study, NCT04350593 should be of particular interest, given the unavailability of any established treatment for the disease (63). Again, overall, the outcomes of these trials may help further optimize blood glucose control strategy for diabetic patients with COVID-19.

**Table 1:** Ongoing trials evaluating the antidiabetic drugs in COVID-19 patients.

**Clinical trial number**	**Clinical phase/Multicenter**	**Arms**	**Target number of patients**	**Primary outcome**	**Estimated date of study completion**
NCT04341935	4/No	• Experimental arm: linagliptin + standard of care insulin regimen as per hospital protocol during hospitalization for up to 14 days • Active comparator arm: standard of care insulin regimen as per hospital protocol during hospitalization for up to 14 days	20	1. Changes in glucose levels	March 30, 2021
NCT04371978	3/No	• Experimental arm: linagliptin + standard of care insulin regimen as per hospital protocol during their entire hospitalization • Nonintervention arm: standard of care insulin regimen as per hospital protocol during their entire hospitalization	100	1. Time to clinical change	September 30, 2021
NCT04365517	3/No	• Active comparator arm: sitagliptin + nutritional therapy with or without insulin treatment • Nonintervention arm: nutritional therapy with or without insulin treatment	170	1. Time for clinical improvement 2. Clinical parameters of acute lung disease 3. Biochemical parameter of acute lung disease	December 30, 2020
NCT04473274	4/No	• Experimental arm: pioglitazone 15–30 mg daily oral or enteral during hospitalization for up to 30 days + standard of care • Standard of care	20	1. Adverse events outcomes without attribution 2. Adverse events attributable	June 1, 2021
NCT04510194	2 (prevention), 3 (treatment)/No	Prevention • Experimental arm: metformin (500 mg; twice daily) • Comparator: placebo Treatment • Experimental: metformin (500 mg; twice daily) • Placebo	1,522	1. Rate of death due to COVID-19 2. Rate of hospitalization due to COVID-19 3. Rate of emergency department utilization 4. Rate of urgent care utilization	September 2021
NCT04350593	3/Yes	• Active comparator: dapagliflozin 10 mg daily • Placebo comparator arm: dapagliflozin matching placebo 10 mg daily	900	1. Time to first occurrence of either death from any cause or new/worsened organ dysfunction through 30 days of follow up	December 2020

## Conclusion

The present review was an attempt to summarize the profiles of currently available antidiabetic agents and their role in maintaining blood glucose control in hospitalized diabetic patients with COVID-19. While it remains important to continue current regimens for glucose control in patients with mild, self-limiting COVID-19, it appears that insulin may be a good choice for patients with severe COVID-19, while DPP4 inhibitors may also prove to be a good choice, along with insulin, for patients with mild to moderate disease, pending the results of clinical trials currently underway. At any rate, given that the COVID-19 pandemic is unlikely to end any time soon and that diabetes is closely associated with disease progression, continued efforts need to be made to accumulate evidence that guides the use of antidiabetic agents in diabetic patients with COVID-19 and to establish the best possible approach to achieving blood glucose control in these patients.

## Author Contributions

HF, MI, MO-I, and TY wrote the manuscript. All authors reviewed the manuscript.

## Conflict of Interest

HF, KO, YA, and YM declare that the research was conducted in the absence of any commercial or financial relationships that could be construed as a potential conflict of interest. MI reports receiving grants from Kowa Pharmaceutical, Merck & Co., Mitsubishi Tanabe Pharma, Nipro Corporation, Ono Pharmaceutical, and Takeda Pharmaceutical outside the submitted work. MO-I reports receiving grants from Boehringer Ingelheim, Kowa Pharmaceutical, Merck & Co., Mitsubishi Tanabe Pharma, Nipro Corporation, Novo Nordisk Pharma Ltd., Ono Pharmaceutical, and Takeda Pharmaceutical outside the submitted work. TK reports receiving personal fees from Abbot Japan, Asahi Mutual Life Insurance Company, AstraZeneca K.K., Bayer AG, Boehringer Ingelheim, Cosmic, FUJIFILM, FUJIREBIO, Johnson & Johnson, Kowa Pharmaceutical, Kyowa Hakko Kirin, Medical Review, Medscape Education, Medtronic Sofamor Danek, Musashino Foods, Nipro Corporation, Novartis International AG, Sanwa Kagaku Kenkyusho, and Terumo Corporation outside the submitted work; grants and personal fees from Astellas Pharma, Daiichi Sankyo, Eli Lilly and Company, Kissei Pharmaceutical, Mitsubishi Tanabe Pharma, Merck & Co., Novo Nordisk Pharma Ltd., Ono Pharmaceutical, Sanofi S.A., Sumitomo Dainippon Pharma, Taisho Pharmaceutical, and Takeda Pharmaceutical outside the submitted work. TY reports receiving grants from AeroSwitch, Asahi Mutual Life Insurance Company, Minophagen Pharmaceutical Co., LTD., Mitsubishi Corporation Life Sciences, Nipro Corporation, NTT DOCOMO, and Tosoh Corporation outside the submitted work; personal fees from Dojindo Molecular Technologies, Eli Lilly and Company, FUJIFILM Toyama Chemical, Johnson & Johnson, Kissei Pharmaceutical, Medtronic Japan (formerly Covidien Japan), and Nippon Becton Dickinson outside the submitted work; grants and personal fees from Astellas Pharma, AstraZeneca K.K., Boehringer Ingelheim, Daiichi Sankyo, Kowa Pharmaceutical, Kyowa Kirin, Merck & Co., Mitsubishi Tanabe Pharma, Novartis International AG, Novo Nordisk Pharma Ltd., Ono Pharmaceutical, Sanofi S.A., Sanwa Kagaku Kenkyusho, Shionogi & Co, Sumitomo Dainippon Pharma, Taisho Pharmaceutical, and Takeda Pharmaceutical outside the submitted work.
